# Genetic analysis of potential biomarkers and therapeutic targets associated with ferroptosis from bronchopulmonary dysplasia

**DOI:** 10.1097/MD.0000000000034371

**Published:** 2023-07-21

**Authors:** Xiaoxue Ma, Ziyu Tao, Leiming Chen, Shaozhi Duan, Guoping Zhou, Yunxia Ma, Zhenqin Xiong, Lan Zhu, Xuejiao Ma, Yan Mao, Yifang Hu, Ni Zeng, Jimei Wang, Yunlei Bao, Fei Luo, Chuyan Wu, Feng Jiang

**Affiliations:** a Department of Neonatology, Yongping County People’s Hospital, Dali, China; b Department of Ultrasound, Obstetrics and Gynecology Hospital of Fudan University, Shanghai, China; c Department of Laboratory Medicine, Obstetrics and Gynecology Hospital of Fudan University, Shanghai, China; d Department of Pediatrics, The First Affiliated Hospital of Nanjing Medical University, Nanjing, China; e Department of Geriatric Endocrinology, The First Affiliated Hospital of Nanjing Medical University, Nanjing, China; f Department of Dermatology, Affiliated Hospital of Zunyi Medical University, Zunyi, China; g Department of Neonatology, Obstetrics and Gynecology Hospital of Fudan University, Shanghai, China; h Department of Rehabilitation Medicine, The First Affiliated Hospital of Nanjing Medical University, Nanjing, China.

**Keywords:** bronchopulmonary dysplasia, diagnostic model, ferroptosis, immune microenvironment, machine learning

## Abstract

Ferroptosis is a recently identified form of cell death that is distinct from the conventional modes such as necrosis, apoptosis, and autophagy. Its role in bronchopulmonary dysplasia (BPD) remains inadequately understood. To address this gap, we obtained BPD-related RNA-seq data and ferroptosis-related genes (FRGs) from the GEO database and FerrDb, respectively. A total of 171 BPD-related differentially expressed ferroptosis-related genes (DE-FRGs) linked to the regulation of autophagy and immune response were identified. Least absolute shrinkage and selection operator and SVM-RFE algorithms identified 23 and 14 genes, respectively, as marker genes. The intersection of these 2 sets yielded 9 genes (ALOX12B, NR1D1, LGMN, IFNA21, MEG3, AKR1C1, CA9, ABCC5, and GALNT14) with acceptable diagnostic capacity. The results of the functional enrichment analysis indicated that these identified marker genes may be involved in the pathogenesis of BPD through the regulation of immune response, cell cycle, and BPD-related pathways. Additionally, we identified 29 drugs that target 5 of the marker genes, which could have potential therapeutic implications. The ceRNA network we constructed revealed a complex regulatory network based on the marker genes, further highlighting their potential roles in BPD. Our findings offer diagnostic potential and insight into the mechanism underlying BPD. Further research is needed to assess its clinical utility.

## 1. Introduction

Bronchopulmonary dysplasia (BPD), also known as neonatal chronic lung disease, is a significant cause of respiratory illness in premature infants and leads to a high incidence of complications and mortality.^[1]^ BPD is a chronic lung disease resulting from impaired and damaged lung development in preterm infants.^[2]^ The diagnostic criterion for BPD is an infant requiring oxygen at 28 days postpartum or at 36 weeks of corrected gestational age. However, this definition does not account for the severity of ultra-prematurity (birth weight <1000 g or gestational age <28 weeks) or respiratory disease.^[3,4]^ Despite its simplicity, this measure is commonly utilized as an outcome indicator for BPD in clinical trials.^[5,6]^ Therefore, there is an immediate need for detectable biomarkers in peripheral blood that can aid in early intervention for BPD.

Ferroptosis is a form of programmed cell death that depends on iron, distinct from apoptosis, necrosis, pyroptosis, and autophagy.^[7,8]^ The process is characterized by mitochondrial atrophy, an increase in mitochondrial membrane density, accumulation of iron and lipid reactive oxygen species, and the involvement of specific genes.^[9–11]^ The presence of circulating iron is crucial for ferroptosis development, and iron chelating agents have effectively in inhibited Erastin-induced ferroptosis.^[12]^ Moreover, the expression of transferrin on the cell membrane can heighten the cell sensitivity to ferroptosis. Despite its novelty, ferroptosis shows promise as a potential therapeutic target for various diseases.

The pathogenesis of BPD is multifactorial, and lacks effective treatments. Complications of BPD often include pulmonary hypertension and right ventricular hypertrophy.^[13,14]^ The pathogenesis of BPD is multifactorial, with prematurity being the most important risk factor. Newborns with abnormal lung structure and function products are particularly susceptible to damage from oxidative stress, free radicals, hypoxia, infection, and other factors.^[15,16]^ Disruption of the oxidative stress balance can trigger various types of programmed cell death, including necrosis, autophagy, pyroptosis, and ferroptosis, all of which have been implicated in the pathogenesis of BPD.^[17–20]^ Among them, ferroptosis has recently gained attention. Understanding the signaling pathways of ferroptosis is critical as this could potentially reveal new therapeutic targets for the clinical treatment of BPD. These studies also help to better understand the molecular and biological mechanisms underlying the progression of BPD. Our research is focused on investigating the pathogenesis of BPD, particularly looking into biomarkers and therapeutic targets associated with iron-dependent cell death in BPD.

## 2. Materials and methods

### 2.1. Data source

**Figure 1.**
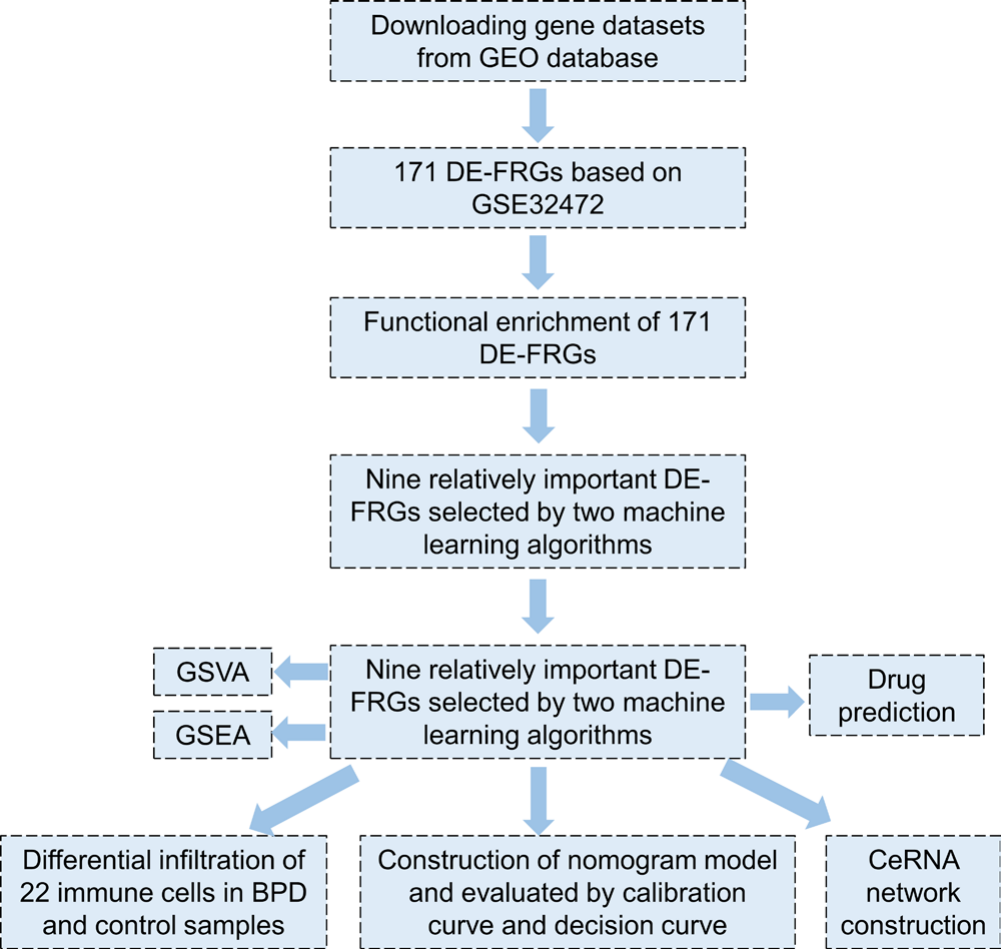
Flow chart.

In this study, we downloaded gene expression datasets of BPD patients and control samples (GSE32472) from the GEO database. The dataset includes gene expression data from blood samples of newborns diagnosed with BPD, with gene expression levels measured at day 5, 14, and 28 after birth. To ensure the stability of the selected samples, we chose 100 blood samples obtained on day 28, by which point BPD diagnosis was relatively clear. Of these samples, 38 were control samples and 62 BPD samples. Additionally, we obtained a list of 420 ferroptosis-related genes (FRG) from the FerrDb database,^[21]^ as detailed in (Table S1, http://links.lww.com/MD/J297, Supplemental Digital Content, 420 genes related to ferroptosis from the FerrDb database). To identify potential therapeutic drugs targeting the marker genes, we analyzed drug-gene interactions using the Drug Gene Interaction database.^[22]^ A schematic diagram of our study is depicted in Figure 1.

### 2.2. Differential expression analysis

Initially, we obtained the expression data for 420 FRGs from both control and BPD samples in GSE32472. Considering genes with a corrected *P*-value below .05 to be statistically significant.

### 2.3. Functional enrichment analysis

We utilized the Metascape database^[23]^ to investigate the potential biological functions of differentially expressed ferroptosis-related genes (DE-FRGs), conducting pathway enrichment analyses, such as gene ontology and kyoto encyclopedia of genes and genomes analyses.

### 2.4. Identification of optimal diagnostic gene biomarkers for BPD

To reduce data dimensionality and identify genetic biomarkers for BPD, we utilized the least absolute shrinkage and selection operator (LASSO) algorithm in the glmnet package, selecting DE-FRGs between BPD patients and control groups.^[24]^ Subsequently, we employed the support vector machine-recursive feature elimination (SVM-RFE) model to further refine our selection of genetic biomarkers for BPD.^[25]^ We identified the intersection of the genes obtained from both the LASSO and SVM-RFE machine learning algorithms. Diagnostic accuracy was evaluated using receiver operating characteristic (ROC) curves. Using 9 selected marker genes, we constructed a diagnostic model and evaluated its performance using ROC curves.

### 2.5. Gene set enrichment analysis (GSEA) analysis

To investigate the pathways associated with the 7 identified marker genes, we conducted a GSEA.^[26]^ This process involved calculating the correlation of the marker genes with all other genes in the GSE32472 dataset, which we then sorted based on their correlation strength. The resulting gene set was then compared with the predefined KEGG signaling pathway set to assess its enrichment. We consolidated the results of each marker gene specific enrichment to infer their potential functions.

### 2.6. GSVA analysis

To study the impact of each marker gene on KEGG pathways, we utilized GSVA analysis.^[27]^ The limma package was used to compare GSVA scores between groups with high and low expression levels of the marker genes. Significance was determined by the criteria of |t| > 2 and *P* < .05. If t > 0, pathways were considered activated in the high-expression group, and if t < 0, pathways were considered activated in the low-expression group.

### 2.7. Immune infiltration analysis

We utilized the CIBERSORT algorithm to calculate the cellular composition based on gene expression profiles. The CIBERSORT algorithm can calculate the proportions of 22 different immune cell types in each sample.^[28]^ It should be noted that the sum of the fractions for all evaluated immune cell types was equal to 1 for each sample.

### 2.8. Construction of ceRNA network

In order to explore the potential regulatory interactions between mRNA and miRNA based on the 6 identified marker genes, we employed the starBase database to predict miRNA sequences in humans and retrieved mRNA-miRNA nucleic acid binding predictions using the miranda software. We then screened miRNA-lncRNA interactions in the starBase database to construct the mRNA-miRNA-lncRNA ceRNA network.

## 3. Results

### 3.1. Identification of DE-FRGs in GSE32472 cohort

**Figure 2.**
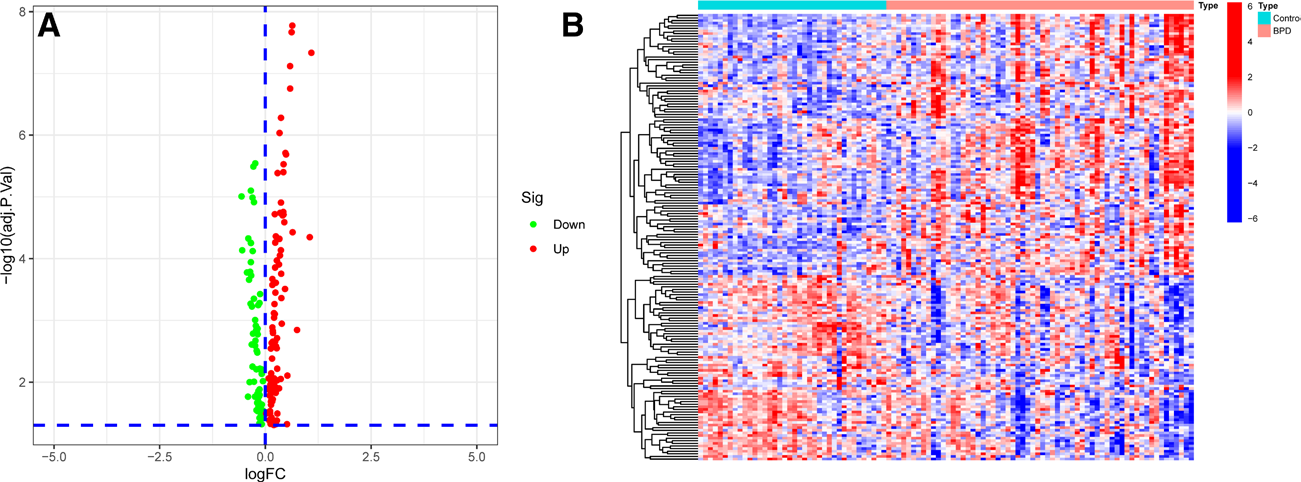
Identification of DE-FRGs of BPD. (A) Volcano plot depicts the results of DE-FRGs. (B) Heatmap of the DE-FRGs. BPD = bronchopulmonary dysplasia, DE-FRGs = differentially expressed ferroptosis-related genes.

According to the screening conditions (|log_2_FC| > 0, *P* value < .05), 171 DE-FRGs were retrieved from the dataset GSE32472 based on R software analysis and screening criteria, including 101 upregulated genes and 70 downregulated genes (Table S2, http://links.lww.com/MD/J298, Supplemental Digital Content, 171 DE-FRGs were retrieved from the dataset GSE32472). A volcano plot (Fig. 2A) was drawn to show the distribution of DE-FRGs in GSE32472 cohort. The hierarchical cluster analysis heat map showed DE-FRGs between BPD samples and control samples (Fig. 2B).

### 3.2. Metascape analysis of DE-FRGs

**Figure 3.**
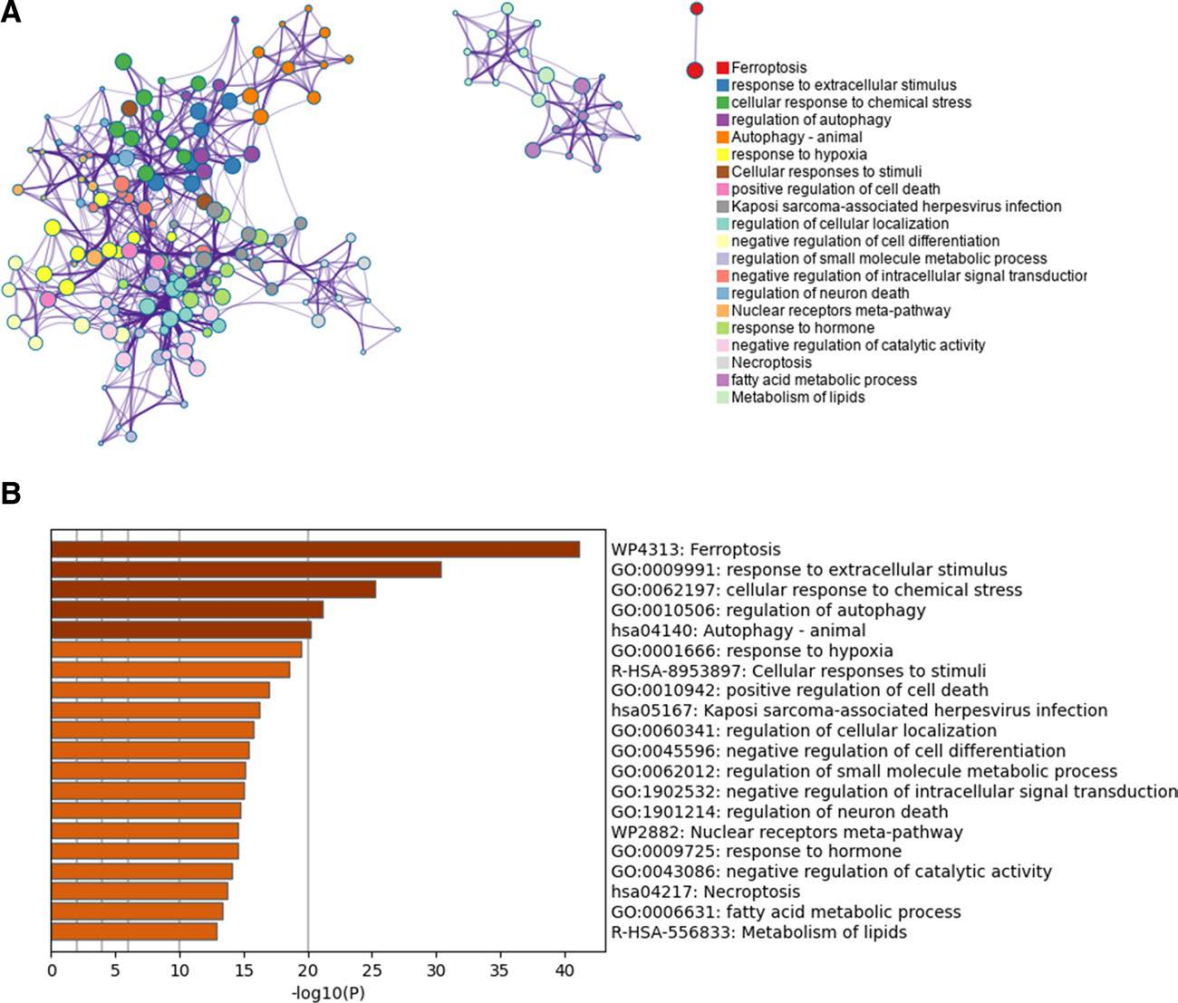
Metascape for GO and KEGG analysis of DE-FRGs in BPD. (A) A network diagram was created with each enriched term as a node, and the similarity between the nodes was represented by the edges. (B) A bar graph was presented, displaying the top 20 enriched terms for biological functions and signaling pathways. BPD = bronchopulmonary dysplasia, DE-FRGs = differentially expressed ferroptosis-related genes, GO = gene ontology, KEGG = kyoto encyclopedia of genes and genomes.

We utilized Metascape to conduct gene ontology and kyoto encyclopedia of genes and genomes pathway analysis on our DE-FRGs, which revealed several significant signaling pathways. As demonstrated in Figure 3, these pathways were associated with ferroptosis, response to extracellular stimulus, cellular response to chemical stress, and regulation of autophagy.

### 3.3. Identification of 9 DE-FRGs as diagnostic genes for BPD

**Figure 4.**
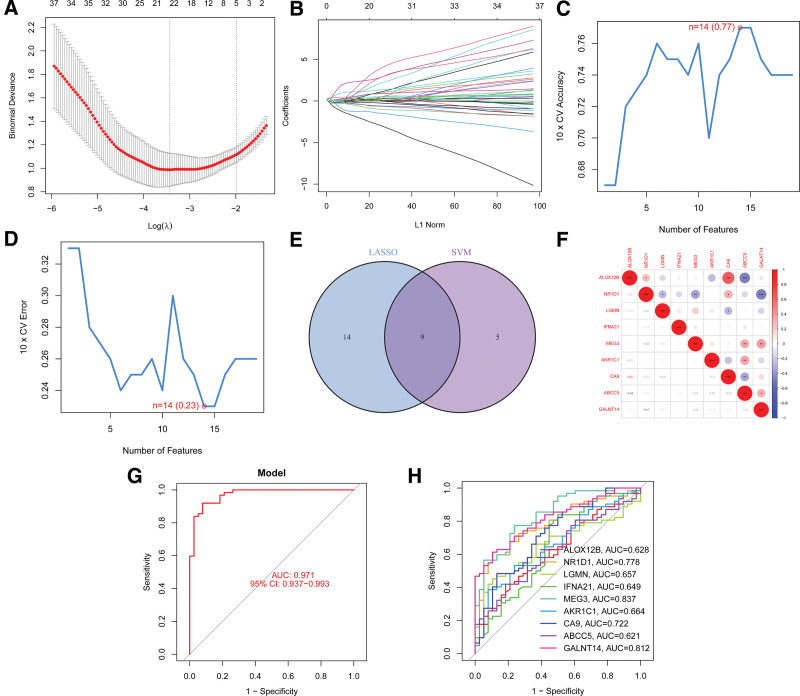
Nine DE-FGs were identified as diagnostic genes for BPD. (A and B) By LASSO logistic regression algorithm, with penalty parameter tuning conducted by 10-fold cross- validation, was used to select 23 BPD- related features. (C and D) SVM-RFE algorithm to filter the 14 DE-FRGs to identify the optimal combination of feature genes. Finally, 9 genes (maximal accuracy = 0.77, minimal RMSE = 0.23) were identified as the optimal feature genes. (E) The marker genes obtained from the LASSO and SVM- RFE models. (F) The correlation between these genes. (G) Logistic regression model to identify the AUC of disease samples. (H) ROC curves for the 9 marker genes. BPD = bronchopulmonary dysplasia, DE-FRGs = differentially expressed ferroptosis-related genes, LASSO = least absolute shrinkage and selection operator, ROC = receiver operating characteristic.

To evaluate the diagnostic potential of DE-FRGs between BPD samples and control samples, we conducted 2 distinct machine learning algorithms (LASSO and SVM-RFE) on the GSE32472 dataset. The objective was to identify significant DE-FRGs that could effectively distinguish BPD from normal samples. The LASSO algorithm was used to select 23 BPD-related features by tuning the penalty parameter via 10-fold cross-validation (as depicted in Fig. 4A and B). We then utilized the SVM-RFE method to screen the 14 DE-FRGs and determine the most effective set of feature genes (as shown in Fig. 4C and D). By analyzing these feature genes further, we discovered 9 marker genes through the intersection of the marker genes obtained from both the LASSO and SVM-RFE models (as depicted in Fig. 4E). The correlation between these genes is displayed in Figure 4F for further evaluation. Interestingly, ALOX12B had a negative correlation with ABCC5. Conversely, ALOX12B was positively correlated with CA9 and NR1D1. However, IFNA21 was not correlated with any DE-FRGs. The correlation analysis of these genes provides further insight into their potential role in the pathogenesis of BPD.

A logistic regression model was constructed using the 9 marker genes identified earlier, with the R package glm. ROC curves generated from the model demonstrated that it was effective in differentiating between control and BPD samples, with an AUC value of 0.971 (as depicted in Fig. 4G). In order to evaluate the discriminatory power of each gene in distinguishing BPD from normal samples, we constructed ROC curves for all 9 marker genes (as illustrated in Fig. 4H). The outcomes indicated that all genes had an AUC value >0.6, indicating their potential in identifying BPD. These findings suggested that the logistic regression model based on the 9 marker genes showed higher accuracy and specificity in differentiating BPD samples from normal samples compared to individual marker genes.

### 3.4. Construction of the nomogram

**Figure 5.**
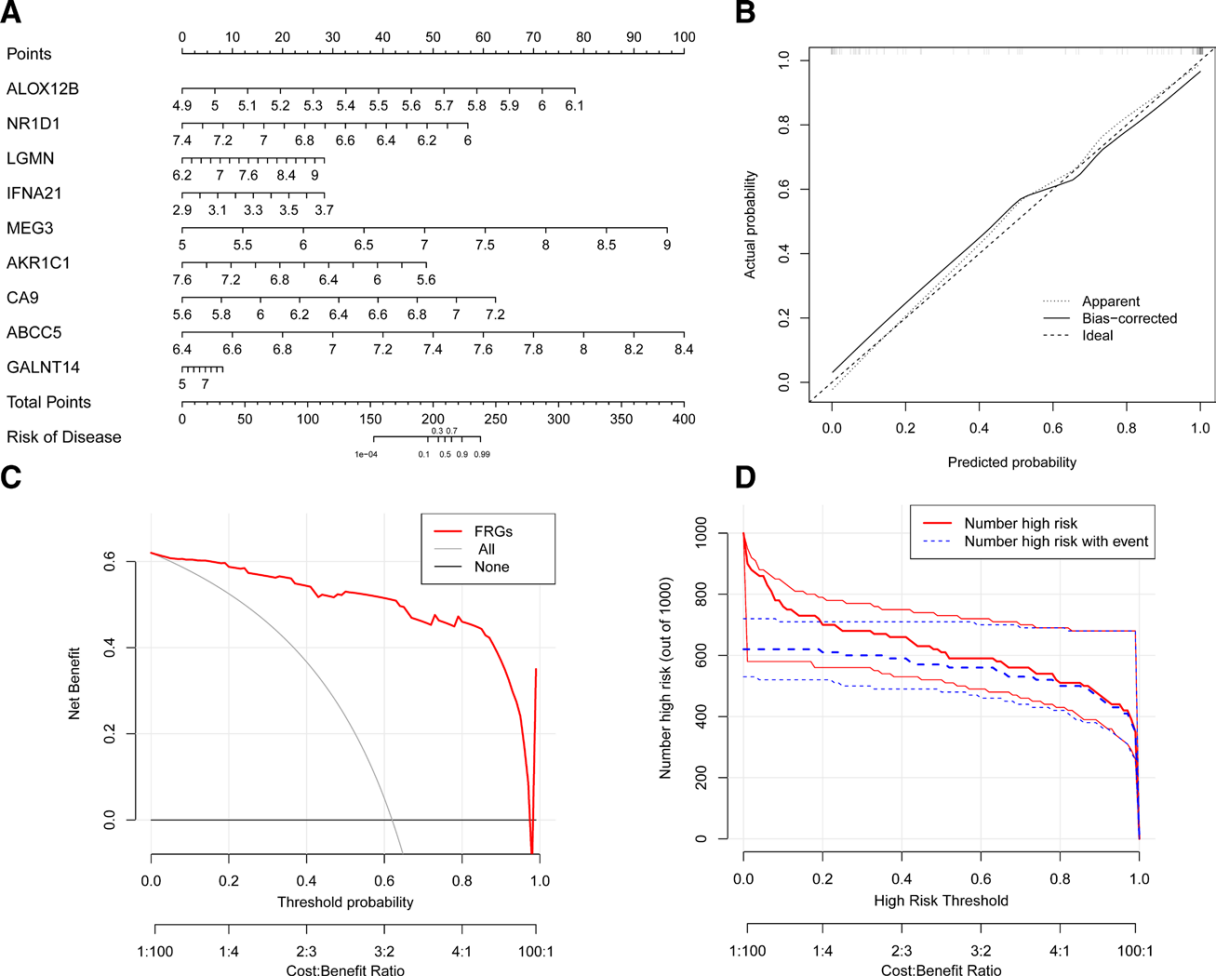
Construction of the nomogram model. (A) Construction of the nomogram model based on the 9 candidate DE-FRGs. (B) Predictive ability of the nomogram model as revealed by the calibration curve. (C) Decisions based on the nomogram model may benefit BPD patients. (D) Clinical impact of the nomogram model as assessed by the clinical impact curve. BPD = bronchopulmonary dysplasia, DE-FRGs = differentially expressed ferroptosis-related genes.

Using the “rms” package in R, we constructed a nomogram model utilizing the 9 candidate DE-FRGs in order to estimate the prevalence of BPD (as presented in Fig. 5A). The calibration curves were employed to evaluate the model accuracy, which indicated that the predictive ability of the model was trustworthy (as indicated in Fig. 5B). To evaluate the clinical significance of the nomogram model, we generated DCA and clinical impact curve plots (Fig. 5C). Additionally, the clinical impact curve plot demonstrated that the nomogram model had exceptional predictive power (as exhibited in Fig. 5D).

### 3.5. Marker genes correlated with multiple BPD-related pathways

**Figure 6.**
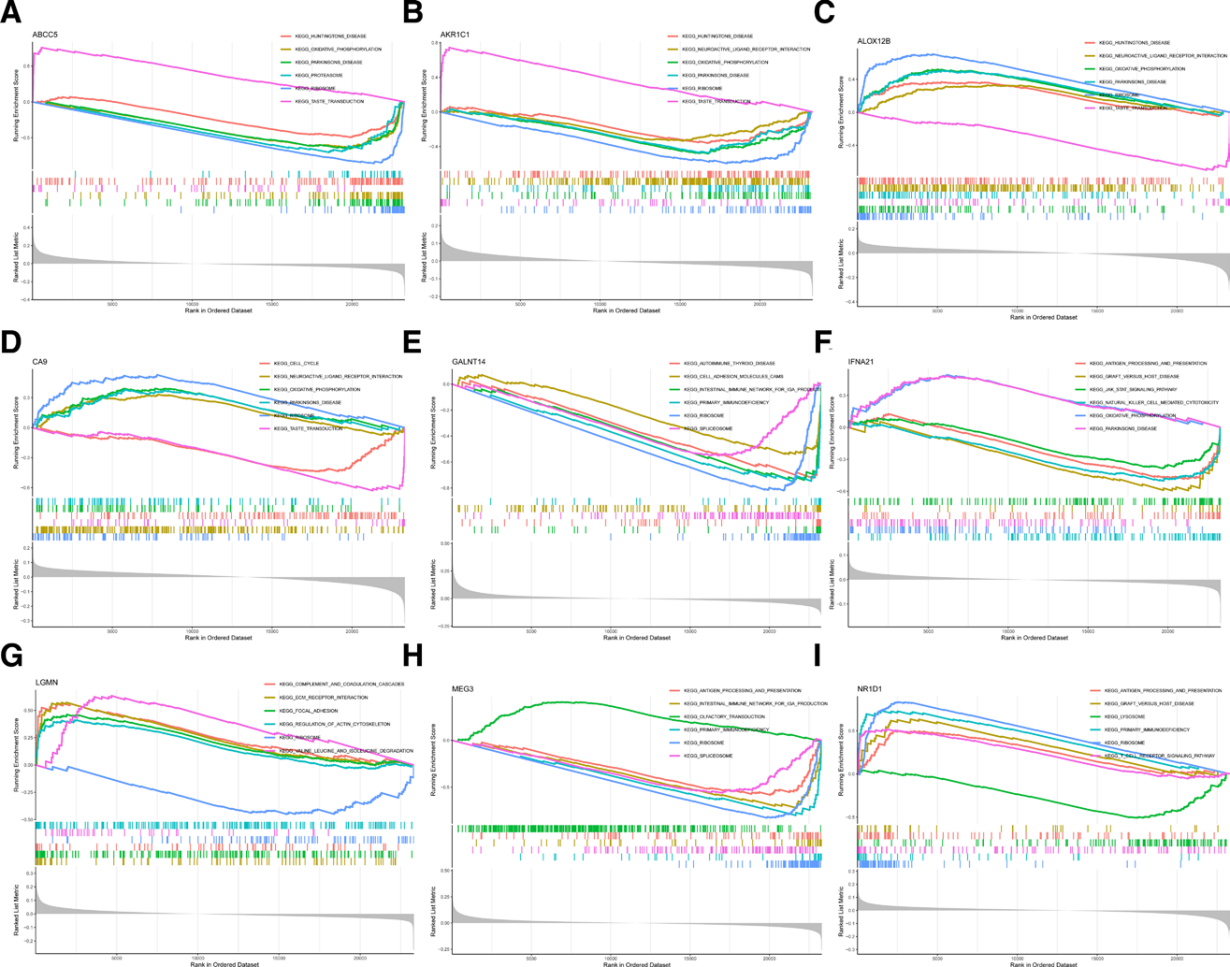
Single-gene GSEA-KEGG pathway analysis in ABCC5 (A), AKR1C1 (B), ALOX12B (C), CA9 (D), GALNT14 (E), IFNA21 (F), LGMN (G), MEG3 (H), and NR1D1 (I). GSEA = gene set enrichment analysis.

To explore the potential role of marker genes in distinguishing normal samples from disease samples, we conducted single-gene GSEA-KEGG pathway analysis. Figure 6A to I display the top 10 pathways enriched by each marker gene. The enriched pathways of these genes included oxidative phosphorylation, ribosome, taste transduction, neuroactive ligand-receptor interaction, cell cycle, and ECM receptor interaction.

**Figure 7.**
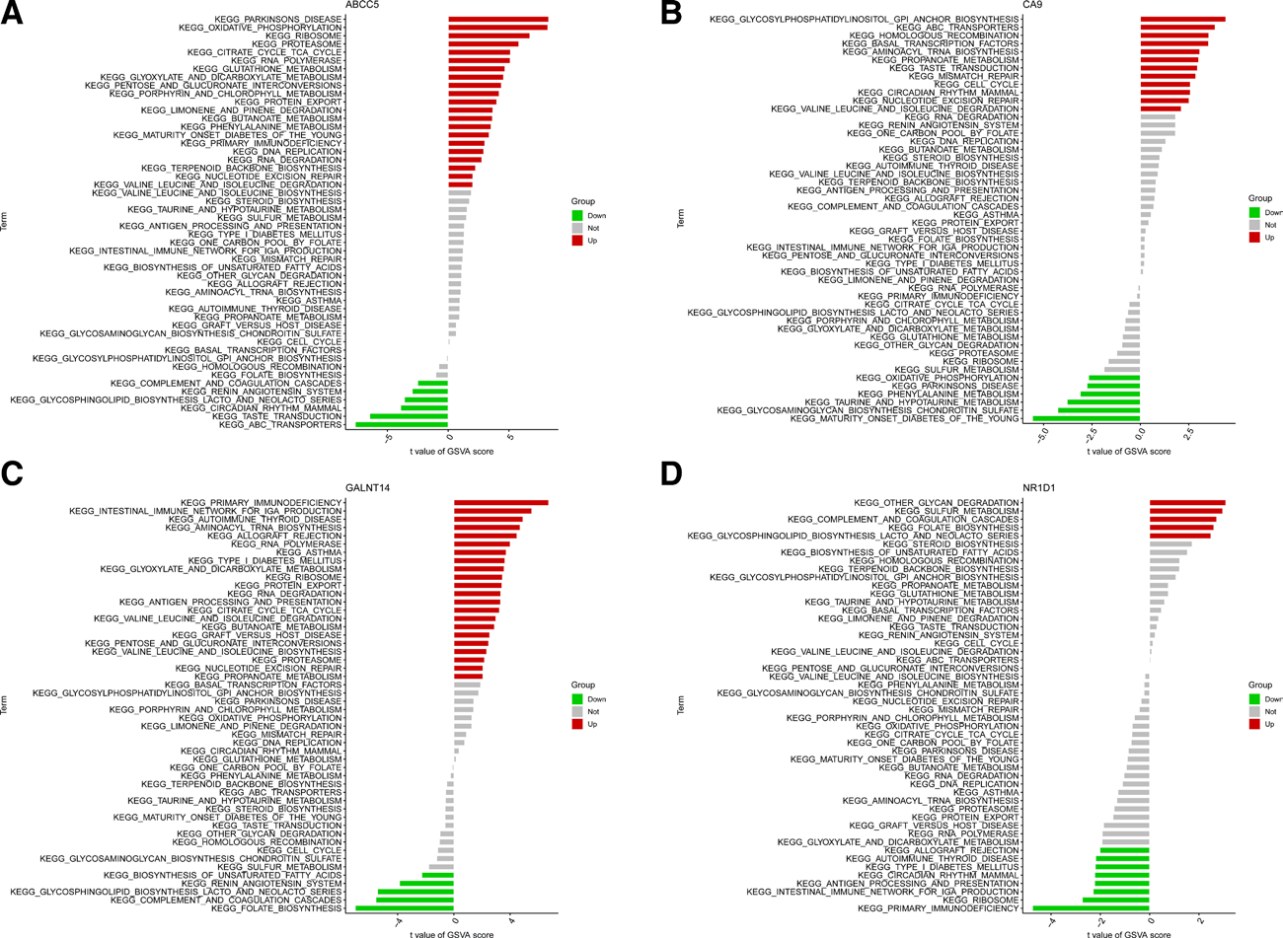
High- and low-expression groups based on the expression levels of each marker gene combined with GSVA in ABCC5 (A), CA9 (B), GALNT14 (C), and NR1D1 (D).

Based on the high or low expression levels of each marker gene, we analyzed the differences in enriched pathways between different expression groups, we used GSVA. Our findings revealed that the high expression of ABCC5 in BPD could potentially lead to BPD by activating oxidative phosphorylation, ribosome, proteasome, citrate cycle TCA cycle while the low expression of ABCC5 activated ABC transporters, taste transduction, and circadian rhythm mammal. High expression of CA9 in BPD may induce BPD by activating glycosylphosphatidylinositol gpi anchor biosynthesis, homologous recombination, and basal transcription factors. Low expression of ABCC5 activated maturity onset diabetes of the young, glycosaminoglycan biosynthesis chondroitin sulfate. High expression of GALNT14 in BPD may induce BPD by activating primary immunodeficiency. Low expression of GALNT14 activated folate biosynthesis. High expression of NR1D1 in BPD may induce BPD by activating other glycan degradation, sulfur metabolism, complement and coagulation cascades. Low expression of NR1D1 activated primary immunodeficiency (Fig. 7A–D).

### 3.6. Immune landscape analysis

**Figure 8.**
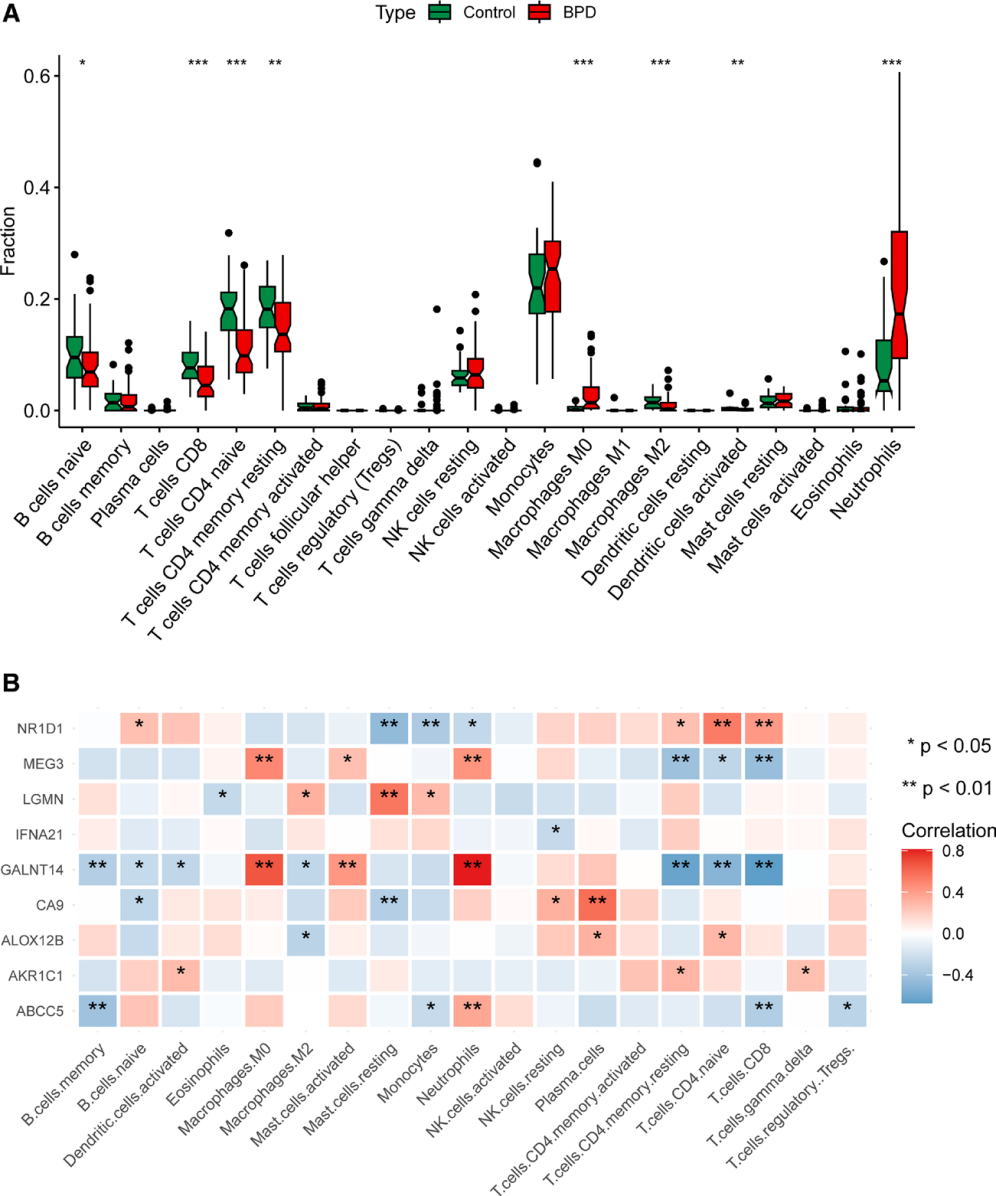
Examination of the immunological landscape of BPD. (A) Correlation analysis of infiltrating immune cells. (B) BPD and control groups were compared on 22 immune-cell subtypes. BPD = bronchopulmonary dysplasia.

Taking into account the outcomes demonstrating the connection between marker genes and immune-related pathways, we employed the CIBERSORT algorithm. The results showed that BPD samples exhibited a decreased proportion of B cells naive, T cells CD8, T cells CD4 naive, and T cells CD4 memory resting compared to control samples, while macrophages M0 and neutrophils were more highly expressed in BPD samples (as indicated in Fig. 8A). Pearson correlation analysis indicated that neutrophils were strongly positively correlated with ABCC5, GALNT14, and MEG3, while macrophages M0 were positively correlated with GALNT14 and MEG3. Moreover, T cells CD4 naive showed positive and negative correlations with GALNT14, MEG3, and NR1D1 (as demonstrated in Fig. 8B). These findings suggest that immune microenvironment in BPD patients may be associated with GALNT14, MEG3, NR1D1, and ABCC5.

### 3.7. Prediction of marker gene-targeted drugs

**Figure 9.**
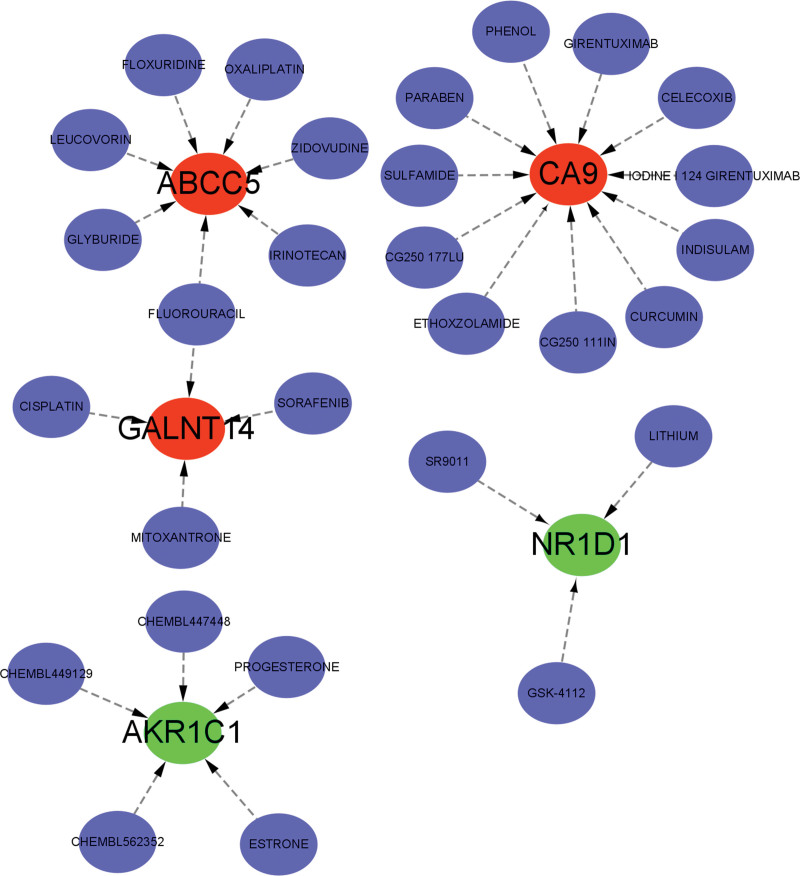
The marker gene-targeted drugs were predicted using the DGIdb database, which identifies drugs that may target these specific genes, and examines their relationship. DGIdb = drug gene interaction database.

Using the Drug Gene Interaction database, we identified potential drugs that could target the marker genes and evaluated the interaction relationships between them utilizing default parameter settings. We employed Cytoscape software to visualize the results (as depicted in Fig. 9). Our analysis revealed 29 drugs that targeted the marker genes, including 7 for ABCC5, 11 for CA9, 4 for GALNT14, 5 for NKR1C1, and 3 for NR1D1. Given that BPD is a chronic inflammatory lung disease with persistent respiratory distress, some of the targeted drugs, such as curcumin and celecoxib, have anti-inflammatory effects. Of note, some of the identified drugs are anti-cancer agents that inhibit RNA formation, such as floxuridine, oxaliplatin, and cisplatin, which are related to ABCC5 and GALNT14. Additionally, progesterone and estrone play an important role in the function of AKR1C1.

### 3.8. A ceRNA network based on marker genes

**Figure 10.**
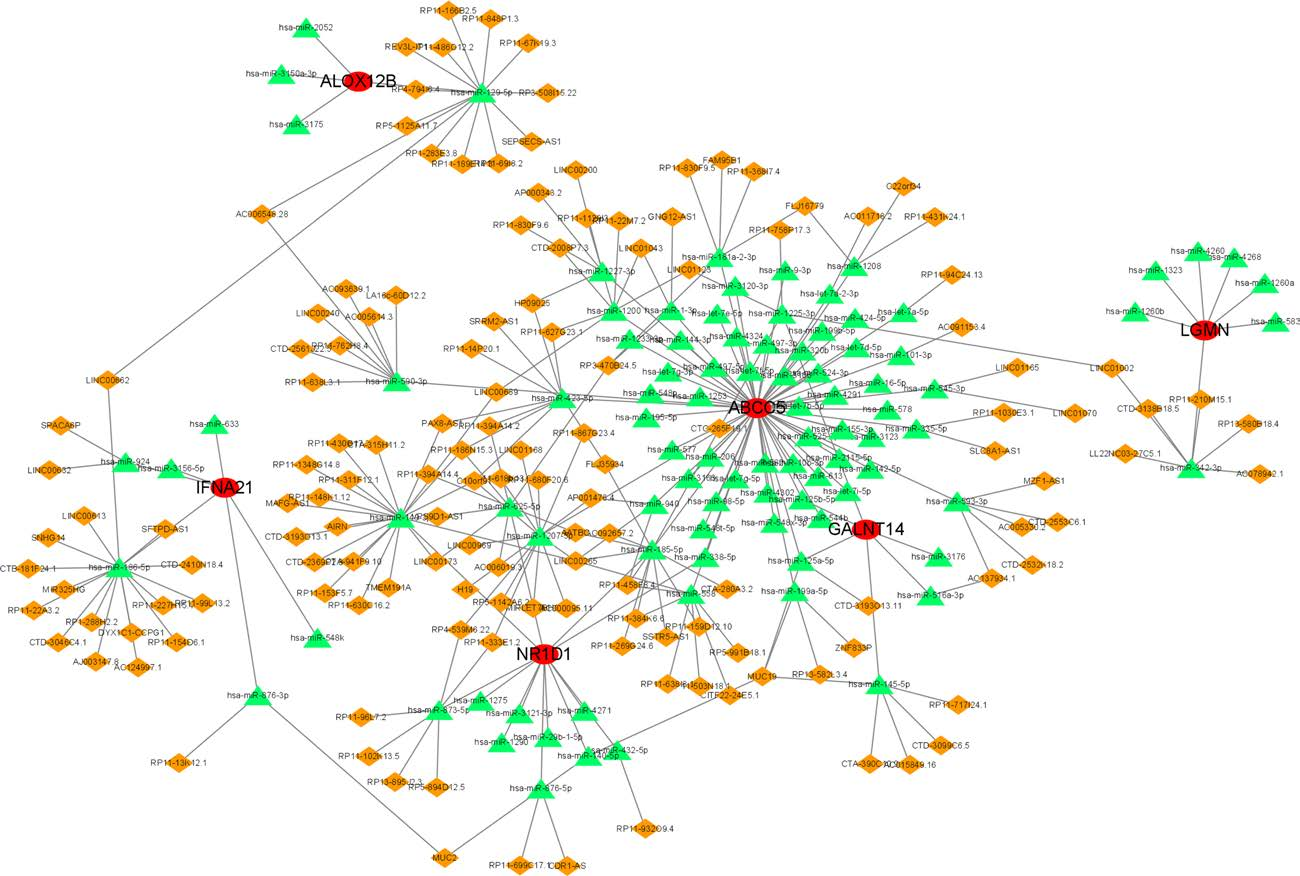
A ceRNA network based on marker genes. The network included 277 nodes (6 marker genes, 98 miRNAs and 179 lncRNAs) and 688 edges.

We constructed a ceRNA network based on 6 marker genes using the starBase and miRanda databases. The resulting network was composed of 277 nodes, including 6 marker genes, 98 miRNAs, and 179 lncRNAs, connected by 224 edges (as presented in Fig. 10). For ABCC5, we identified 62 mRNAs that could regulate its expression by competitively binding with hsa-miR-320b, thus exerting a regulatory effect on this gene. In the LGMN ceRNA network, we identified 7 mRNAs that could combine with hsa-miR-1323, hsa-miR-524-3p, and hsa-miR-3166 to regulate the gene, and 12 mRNAs that could regulate the expression of NR1D1 by competitively binding with hsa-miR-873-5p. Furthermore, we identified 6 common mRNAs that targeted hsa-miR-125a-5p, hsa-miR-144-3p, hsa-miR-558, hsa-let-7f-5p, hsa-miR-155-3p, and hsa-miR-940, respectively, which influenced the expression of GALNT14. These findings suggest a complex network of interactions between mRNA, miRNA, and lncRNA in BPD development, with these 6 marker genes playing important roles in the process.

## 4. Discussion

Despite significant advances in perinatal healthcare over the last 2 decades, including a decline in very low birth weight deaths, BPD remains a major complication of preterm birth and a significant cause of long-term complications.^[29,30]^ Preterm birth and low birth weight continue to be the major risk factors for BPD. As BPD is a chronic disease with multiple causes, many infants and children with BPD require comprehensive management and multidisciplinary treatment long after the age of 1. Although BPD tends to improve with age, it can have lifelong effects.

It should be noted that the association between BPD and ferroptosis has not been previously documented. Therefore, to explore the molecular pathogenesis of BPD ferroptosis, it was important to select appropriate gene chip data, perform gene expression data analysis, and use bioinformatics methods such as single-gene analysis and enrichment to find common points and analyze genetic differences in BPD and control samples. Our study employed a thorough selection of gene chips, utilized multiple gene samples, and analyzed a vast amount of microarray data to ensure the precision and dependability of our findings. The outcomes of this study can serve as a valuable clinical resource for the prevention and treatment of BPD.

In this study, we identified 9 differential genes related to ferroptosis, including ALOX12B, NR1D1, LGMN, IFNA21, MEG3, AKR1C1, CA9, ABCC5, and GALNT14. The AUC for all 9 genes was found to be >0.6, suggesting that they possess considerable accuracy and specificity in distinguishing BPD samples from normal samples. Among them, MEG3, GALNT14, and NR1D1 had the top 3 AUC values. The GALNT family is an initiating enzyme that catalyzes the O-glycosylation modification of mucins, and its ability to affect the O-glycosylation of mucins can impact tumor cell genesis, prognosis, proliferation, and migration.^[31,32]^ GALNT14 is a newly discovered member of this family, and recent studies have found that it is abnormally expressed in various tumors and chronic inflammatory diseases, and is associated with tumor cells, inflammatory factors, the development of invasion, metastasis, apoptosis, and ferroptosis, among others.^[33–36]^ BPD is a chronic lung disease of prematurity characterized by fibrosis, emphysema, and chronic inflammation of the lungs. Thus, GALNT14 plays an important role in the progression of ferroptosis, and its expression is more likely to be associated with respiratory enzyme activity in the chronic inflammatory expression of BPD.

The lung immune microenvironment is vital in maintaining lung homeostasis, with a range of immune cells, including macrophages, monocytes, neutrophils, dendritic cells, T and B cells, eosinophils, and mast cells. These cells are involved in injury repair and are essential for lung function. Our analysis showed that the BPD group had higher expression of T-cell helper and neutrophils while showing lower levels of B-cell memory compared to the normal group. Additionally, we found a positive correlation between GALNT14 and neutrophils, which belongs to the polypeptide N-acetylgalactosamine aminotransferase protein family, and its expression is crucial in chronic inflammation. Conversely, NR1D1 was negatively correlated with neutrophils and had decreased expression in BPD patients. Therefore, NR1D1 might be a potential target for improving chronic lung inflammation in BPD patients.

Finally, we conducted an analysis of gene-targeted drugs for marker genes and a ceRNA network. Among the 11 CA9-targeted drugs that we retrieved, variant activator of CBS has been shown to be effective. Celecoxib is a non-steroidal anti-inflammatory drug that has been used to treat diseases such as osteoarthritis, but its efficacy in treating chronic inflammation of the lung has not been reported. Paraben is an organic compound that works by disrupting the cell membrane of microorganisms, denaturing intracellular proteins, and inhibiting the activity of respiratory and electron transfer enzyme systems of microbial cells. The therapeutic effectiveness of the gene-targeted drugs and non-coding RNA that we predicted remains uncertain, and further investigation is needed to determine the specific pathways through which they exert their effects. As such, the drugs and non-coding RNA selected in this study require prospective analysis to better comprehend their potential therapeutic value.

We have identified 9 genes that are potentially involved in ferroptosis in BPD samples. However, these genes are not limited to their association with ferroptosis. The identified biomarkers in this study may be involved in regulating chronic lung inflammation in BPD patients. While gene expression does not always indicate protein expression, the importance of these identified biomarkers should not be underestimated, and their research significance should be acknowledged. Our ongoing research will delve deeper into these genes to expand our understanding of the pathogenesis and treatment of BPD.

## 5. Conclusions

In summary, we have identified 273 significant targets in BPD patients. Using 3 different machine learning algorithms, we have determined that CYYR1, GALNT14, and OLAH have potential diagnostic value as biomarkers in peripheral blood for BPD. Further research on the underlying mechanisms of these genes may lead to new insights for the prevention, diagnosis, and treatment of BPD. Nevertheless, the precise action mechanisms of these 3 genes in the development and progression of BPD require further investigation.

## Author contributions

**Conceptualization:** Fei Luo, Feng Jiang.

**Data curation:** Lan Zhu, Fei Luo.

**Formal analysis:** Xiaoxue Ma, Yunxia Ma.

**Investigation:** Zhenqin Xiong, Yunlei Bao, Feng Jiang.

**Methodology:** Ziyu Tao, Jimei Wang, Yunlei Bao.

**Project administration:** Xuejiao Ma.

**Resources:** Chuyan Wu.

**Software:** Leiming Chen, Shaozhi Duan, Ni Zeng.

**Validation:** Yan Mao, Yifang Hu.

**Visualization:** Guoping Zhou, Jimei Wang.

**Writing-original draft:** Xiaoxue Ma.

**Writing-review & editing:** Feng Jiang.

## Supplementary Material
